# Estimates of effectiveness and reach for ‘return on investment’ modelling of smoking cessation interventions using data from England

**DOI:** 10.1111/add.14006

**Published:** 2017-09-14

**Authors:** Robert West, Kathryn Coyle, Lesley Owen, Doug Coyle, Subhash Pokhrel, On behalf of the EQUIPT Study Group

**Affiliations:** ^1^ Department of Behavioural Science and Health University College London London UK; ^2^ Health Economics Research Group (HERG), Institute of Environment, Health and Society Brunel University London Uxbridge UK; ^3^ Centre for Guidelines National Institute for Health and Care Excellence London UK; ^4^ School of Epidemiology and Public Health University of Ottawa Ottawa Canada

**Keywords:** Effectiveness, models, quit attempt, reach, return on investment, smoking

## Abstract

**Background and aims:**

Estimating ‘return on investment’ (ROI) from smoking cessation interventions requires reach and effectiveness parameters for interventions for use in economic models such as the EQUIPT ROI tool (http://roi.equipt.eu). This paper describes the derivation of these parameter estimates for England that can be adapted to create ROI models for use by other countries.

**Methods:**

Estimates were derived for interventions in terms of their reach and effectiveness in: (1) promoting quit attempts and (2) improving the success of quit attempts (abstinence for at least 12 months). The sources were systematic reviews of efficacy supplemented by individual effectiveness evaluations and national surveys.

**Findings:**

Quit attempt rates were estimated to be increased by the following percentages (with reach in parentheses): 20% by tax increases raising the cost of smoking 5% above the cost of living index (100%); 10% by enforced comprehensive indoor public smoking bans (100%); 3% by mass media campaigns achieving 400 gross rating points (100%); 40% by brief opportunistic physician advice (21%); and 110% by use of a licensed nicotine product to reduce cigarette consumption (12%). Quit success rates were estimated to be increased by the following ratios: 60% by single‐form nicotine replacement therapy (NRT) (5%); 114% by NRT patch plus a faster‐acting NRT (2%);124% by prescribed varenicline (5%); 60% by bupropion (1%); 100% by nortriptyline (0%), 10) 298% by cytisine (0%); 40% by individual face‐to‐face behavioural support (2%); 37% by telephone support (0.5%); 88% by group behavioural support (1%); 63% by text messaging (0.5%); and 19% by printed self‐help materials (1%). There was insufficient evidence to obtain reliable, country‐specific estimates for interventions such as websites, smartphone applications and e‐cigarettes.

**Conclusions:**

Tax increases, indoor smoking bans, brief opportunistic physician advice and use of nicotine replacement therapy (NRT) for smoking reduction can all increase population quit attempt rates. Quit success rates can be increased by provision of NRT, varenicline, bupropion, nortriptyline, cytisine and behavioural support delivered through a variety of modalities. Parameter estimates for the effectiveness and reach of these interventions can contribute to return on investment estimates in support of national or regional policy decisions.

## Introduction

‘Return on investment’ (ROI) estimates for public health interventions can inform resource allocation decisions usefully at national or regional levels 1. They are dependent upon estimates of the effectiveness and reach of the interventions being evaluated. Interventions to promote smoking cessation are recognized as particularly important in public health 2. They include a range of approaches from education and persuasion to restrictions and support services 3. This paper describes the processes and outcomes of an exercise to arrive at reach and effect‐size estimates for cigarette smoking cessation interventions undertaken to inform development of a Europe‐wide ROI calculation tool (EQUIPT) 4. Cigarettes are the most commonly used tobacco product and the one for which there is the most evidence, so the scope of this review is limited to cigarette smoking.

Developing a strategy to promote smoking cessation can be based usefully on an evaluation of the probable impact of the components of that strategy relative to their cost. Cost‐effectiveness (CE) estimation is usually expressed in terms of cost per ‘quality‐adjusted’ life‐year gained, ‘disability‐adjusted’ life‐year gained or sometimes simply life‐year gained 5. In the case of smoking cessation there are financial savings; for example, from not having to treat smoking‐related illnesses, and from less time lost to sickness absence and time off work for cigarette breaks 6. This means that one can go further than estimating cost‐effectiveness from a health service perspective to making a judgement about ‘cost–benefit’ from a wider perspective (CB) 5. When it comes to developing policies, CB estimates can then be used to estimate the overall ‘return on investment’ (ROI) 7: the economic benefit for a given jurisdiction such as a region or country over a given time‐scale. This will depend upon the size of the population, the prevalence of smoking and the population's demographic characteristics.

Both CB, and therefore ROI, estimates depend upon assumptions about the effects of the interventions in promoting smoking cessation in a real‐world setting. Effectiveness of behaviour change interventions is often context‐sensitive and depends upon specific features of those interventions 8. This means that effect‐size estimates from studies in the research literature require a set of assumptions in order to be translated into effectiveness estimates in populations. A systematic process needs to be adopted for this, and the resulting estimates need to be qualified accordingly to ensure their fitness for the specific purpose of any research endeavour.

ROI estimates also depend upon the reach of interventions. A highly effective intervention that reaches a small only proportion of the target population may have a lower overall ‘impact’ (reach × effectiveness) than an intervention with a small effect but to which a large proportion of the population is exposed 9. Reach can also be important for the cost part of CB estimation. Some interventions, such as mass media campaigns, have substantial fixed costs and so may be highly cost‐beneficial when reach is high, but not if reach is low.

Effectiveness and reach of smoking cessation interventions have been estimated in several reviews, and the findings incorporated into CB or ROI estimation exercises 10, 11, 12, 13, 14, 15, 16, 17, 18, 19, 20, 21, 22. However, as evidence accumulates we can make improved estimates for interventions already known about, add estimates for new interventions or new versions of interventions and revise estimates for interventions to take account of varying context.

The proportion of a population that stops smoking in a given time‐period is the proportion who try to quit multiplied by the proportion of those who maintain abstinence 23. These two parts of the process involve different mechanisms and are subject to different modifiers 24. Different interventions may have different effects on these two parts of the process of cessation, so modelling the impact of smoking cessation interventions needs to recognize this. Therefore, parameter estimates for effectiveness and reach of smoking cessation interventions need to relate separately to these two components of cessation.

Through the Smoking Toolkit Study (STS) 25, England has unique data on large, nationally representative samples of smokers and recent ex‐smokers that are updated every month. The STS provides real‐world estimates of effectiveness of interventions on quit attempts and quit success as well as up‐to‐date estimates of reach in a country that has a strong tobacco control climate. The effect‐size estimates can be used to check how far findings from randomized controlled trials (RCTs) or other experimental studies translate into a national setting, and the reach estimates can provide a benchmark for modelling in countries for which such data are not available.

This paper reports the process used to arrive at up‐to‐date effectiveness (in terms of quit attempts and quit success) and reach estimates for smoking cessation interventions, using England as a case example. It presents the estimates relating to a wide range of interventions together with rationales and caveats. The parameters derived from this process have been incorporated into a spreadsheet and combined with cost and health outcome data to generate ROI estimates in EQUIPT. Any of the parameters are potentially modifiable, so that individual countries can reflect their own local data and circumstances.

## Methods

We first identified interventions that were judged suitable for inclusion and specified versions of these for which effect‐size estimates could be derived. We then assessed the reach of those interventions in England, and finally carried out effectiveness estimation.

### Identification of interventions to be evaluated

We first identified broad types of smoking cessation interventions for which it was judged to be possible to arrive at effectiveness estimates. Effectiveness estimates can only be derived from: (1) randomized controlled trials (RCTs), (2) quasi‐experimental studies, (3) comparative observational studies or, in the case of population‐level interventions, (4) time–series analyses. We excluded interventions in which evidence was judged insufficient for confident effect‐size estimation at the time of writing (e.g. e‐cigarettes with only two small RCTs and one large real‐world study, standardized packing where it was too early to be able to estimate an effect with confidence, nicotine replacement therapy pre‐loading where results are not sufficiently conclusive, internet applications where the results show large unexplained heterogeneity and smartphone applications where we lack published RCTs of sufficient power).

The lead author had recently undertaken a major review of clinical interventions that combined evidence, primarily from Cochrane Reviews acknowledged to be of high quality, with additional RCT evidence and evidence from real‐world evaluations being checked to confirm the estimates from the reviews 23. It included screening clinical interventions for those that were effective and where the effect size could be estimated.

For population‐level interventions, we began by searching for those that had been reviewed by Cochrane, and then undertook a review of reviews to assess whether there were any additional interventions that could be included. This involved searching Pubmed and Web of Science using the terms ‘Tobacco control’ + ‘intervention’ + ‘review’. We identified reviews from which we could derive effect‐size estimates for quit attempts or quit success.

We excluded reviews that focused on: (1) specific subgroups such as women, smokers with psychiatric disorders (e.g. 26) or smokers with substance use disorders (because of lack of evidence for differential effectiveness); (2) outcomes other than overall effect sizes (e.g. health inequalities 27, 28); (3) particular behavioural techniques, such as motivational interviewing 29; and (4) smoking initiation 30.

Having identified broad types of smoking cessation interventions, we then sought to characterize a particular variant of that intervention for use in the effectiveness and reach estimation. Thus, any of the interventions could take a wide variety forms in terms of the way they are delivered and their intensity. For example, increasing tax on tobacco products can be undertaken in many ways and by varying amounts. Given that the effect depends critically upon this translating into an increase in the financial cost of smoking, it is essential to capture this feature of the tax increase in the description of the intervention. Similarly, mass media campaigns can take different forms and to very different levels of intensity. We needed to arrive at a specific case that could be used for modelling purposes.

The process for arriving at specific variants of the intervention types involved analysing the key ingredients of the intervention and the typical level of intensity, and specifying this at a level of descriptive detail that could guide the development of new instances of that intervention type. For each intervention type, the rationale for specifying a given variant and the caveats attached to the choices made are reported in this paper.

### Reach estimation

Reach of the interventions was estimated using data from the Smoking Toolkit Study 25, in the case of interventions that required health professionals to deliver them, or smokers to engage actively with them. These estimates are made available on a monthly basis at Smoking in England website (http://www.smokinginengland.info). We used figures for 2016 published on the website.

In the case of population‐level measures it was assumed to be 100%. This did not mean that we assumed that all smokers would be exposed to the intervention, but rather that the effect‐size estimate already captured the extent to which reach may have been less than 100%. For example, tax increases leading to a given increase on the cost of smoking may not have done so for all smokers because of cost‐minimizing strategies, such as switching to hand‐rolled tobacco, reducing the number of cigarettes consumed or purchasing from illicit sources. However, the effect size from studies will have already taken into account those strategies as they were enacted at the time.

### Effectiveness estimation

For the purpose of ROI modelling, effectiveness estimation needed to be expressed using a single metric that could be applied to varying contexts. Given that the outcomes (making an attempt to stop and success of that attempt) were binary, many of the reviews expressed effects in terms of odds ratios. Quit attempts have been defined variously in the research literature, but refer generally to a serious attempt to stop smoking permanently. In some studies, the definition also requires at least 24 hours of abstinence. Quit success is also subject to multiple definitions. Given that ROI modelling assumes that abstinence is permanent, the outcome measure needs to be predictive of lasting abstinence. Longitudinal studies of smoking cessation have shown that 12 months of continuous abstinence provides a reasonably accurate estimation of permanent cessation, with approximately 70% of those who are abstinent for 12 months managing to maintain this for many years 31. Most of the studies included in Cochrane Reviews use 12 months as the final follow‐up point. For comprehensibility and to ease the combination of attempt rates and success rates to arrive at overall cessation rates, we converted odds ratios (OR) where they were used to relative risks (RR) using the formula RR = OR/[(1–*P*) + (*P* × OR)], where *P* is the probability of the outcome of interest in the non‐exposed/control group, obtained from the source that provided the OR.

In the case of the clinical interventions, we were able to draw upon the methods and findings from the review undertaken previously by the lead author 23. This review started with efficacy estimates from Cochrane Reviews and assessed how far evidence from real‐world studies could be expected to translate into outcomes in routine clinical practice. We sought out the most recent reviews in order to update the estimates or, if reviews were not available, the most recent individual studies.

In the case of the population‐level interventions, we started with estimates from the most recent reviews and compared these with findings from earlier ones to assess the stability of the estimates. In some cases, the reviews did not differentiate between effects on quit attempts and quit success. In those cases we sought out studies that did make this differentiation. If there were none, we made an assumption that all the effect was on one or the other. This assumption may not have been correct (e.g. there may have been an effect on both attempt and success rates), but this would not have an impact on the overall cessation rates produced by combining attempt and success rate in the ROI model as it was derived from those overall cessation rates.

## Results

Table 1 gives the specification of the interventions included in the review and the justification for the specification adopted. In all cases they are included because there is sufficient evidence to be able to make an effect‐size estimation. The interventions are classified according to whether they are modelled as increasing the quit attempt rate or the quit success rate.

**Table 1:** Interventions included in the analysis.

Intervention	Specification	Justification for specification
**(a) Interventions to increase the quit attempt rate**		
Increases in taxation	Increasing taxation and implementing countermeasures to prevent illicit supply resulting in an increase in the average cost of smoking 5% above the cost of living index	Increasing taxation by itself need not lead to a rise in the cost of smoking because of multiple methods of mitigating the impact, and the evidence specifically focuses on the financial cost of smoking, not on taxation on its own
Ban on smoking in indoor public areas	A comprehensive ban on smoking in all indoor public areas, including bars, together with mass media campaigns and enforcement to ensure near 100% compliance	The evidence base relates to bans of this kind. Partial bans or bans that are not complied with appear to have little or no impact
Mass media campaigns	Provision of verbal messaging and imagery about smoking and stopping smoking constructed in accordance with principles set out in Public Health England communication strategy document or equivalent; sufficient activity, primarily TV, to accumulate 400 gross rating points (a standard measure of average per‐capita advertising exposure commonly used in evaluations of televised campaigns combining reach and frequency); between 4 and 10 weeks during the year	Evidence suggests that mass media campaigns need to be a minimum intensity and sustained over a minimum period in order to have a detectable effect. The term ‘social marketing’ is used in the EQUIPT model but it should be noted that social marketing (e.g. use of social media) that goes beyond mass media campaigns has not been evaluated adequately
Brief physician advice	Provision of advice to stop smoking with discussion about the best available options for stopping according to principles set out in NCSCT brief advice training; taking up to 5 minutes; delivered by physician trained to NCSCT standard; provided opportunistically to all smokers attending the surgery at least once a year	Evidence suggests that brief opportunistic advice including offer of support has a greater effect than advice alone, and need only take a few minutes
NRT for ‘reduce to quit’	Provision of NRT to smokers interested in stopping smoking but not willing to quit within the next few weeks; to support them to reduce their smoking with a view to quitting in the succeeding weeks	The RCTs on which this intervention description is based included smokers who were motivated to quit but not within the next few months
**(b) Interventions to increase success of quit attempts**		
Single‐form NRT	Provision of one of the many forms of NRT (chewing gum, transdermal patch, lozenge, sublingual tablet, nasal spray, inhalator, mouth spray); typically enough to deliver > 1 mg nicotine per hour systematically; starting on the target quit date and continuing for 8 weeks; provided in person by health professional, retailer or by post; instructed by health professional on use, effects and side effects; free or minimal cost to user; used by smokers of at least 10 cigarettes per day making a quit attempt	The specification describes the intervention as it has been assessed in RCTs. No clear difference has been found between different forms of NRT. Evidence from real‐world studies suggests that NRT may not be effective if bought from a shop without any health professional being involved or additional materials provided 32
Dual form NRT	Provision of nicotine transdermal patch together with one of the faster acting forms; typically daily patch plus *ad lib* use of additional product; starting on the target quit date and continuing for 8 weeks; delivered in person by health professional, retailer or by post with instruction by health professional on use, effects and side‐effects; free or minimal cost to user; used by smokers of at least 10 cigarettes per day making a quit attempt	The specification describes the intervention as it has been assessed in RCTs. Evidence from real‐world studies suggests that NRT may not be effective if bought from a shop without any health professional being involved or additional materials provided 32
Varenicline (Champix)	Provision of varenicline (Champix) 0.5 mg twice daily for 1 week then 1 mg twice daily for 11 weeks; starting at least 1 week prior to target quit date with a total treatment duration of 12 weeks; delivered by health professional on prescription with instruction by health professional on use, effects and side‐effects; free or minimal cost to user; used by smokers of at least 10 cigarettes per day making a quit attempt	The specification describes the intervention as it has been delivered in RCTs. The effect size is confirmed by real‐world studies 33, 34
Varenicline (extended duration)	As above but provided for 24 weeks instead of 12	The specification describes the intervention as it has been delivered in a large RCT 35
Bupropion (Zyban)	Provision of bupropion 150 mg once daily for 6 days, then 150 mg twice daily for 6–8 weeks; starting 1–2 weeks prior to target quit date with a total treatment duration of 7–9 weeks; delivered in person by health professional on prescription; with instruction by health professional on use, effects and side effects; free or minimal cost to user; used by smokers of at least 10 cigarettes per day making a quit attempt	The specification describes the intervention as it has been delivered in RCTs
Nortriptyline	Provision of nortriptyline (generic) 75–100 mg per day titrated to therapeutic levels for depression using serum concentrations; starting 1–2 weeks prior to target quit rate with total treatment duration of 12–14 weeks; delivered in person by health professional on prescription with instruction by health professional on use, effects and side effects; free or minimal cost to user; used by smokers of at least 10 cigarettes per day making a quit attempt	The specification describes the intervention as it has been delivered in RCTs
Cytisine	Provision of cytisine (generic; available brands: Tabex and Desmoxan) 100 1.5 mg tablets in total; six tablets per day for 3 days, then 5 tablets per day for 9 days, then four tablets per day for 4 days, then three tablets per day for 4 days, then one to two tablets per day for 5 days; starting up to 1 week before target quit date and continuing for 25 days; delivered in person by health professional on prescription with instruction by health professional on use, effects and side effects; free or minimal cost to user; used by smokers of at least 10 cigarettes per day making a quit attempt	The specification describes the intervention as it has been delivered in RCTs
Behavioural support (face‐to‐face, individual)	Provision of practical advice and emotional support and encouragement based on Maudsley model; typically weekly sessions, 1 h for first session then approximately 30 minutes on average after that; usually starting 2 weeks before the quit date and continuing for at least 4 weeks afterwards; delivered in person by a health professional trained to NCSCT standard or equivalent; provided in an office or clinic setting in‐person by a single practitioner to a single client or patient; premises, equipment and infrastructure support and supervision for practitioner, including ongoing monitoring of outcomes as per Public Health England Service and Monitoring Guidance or equivalent; free or low cost to user; used by smokers making a quit attempt during the year	The specification is derived from analysis of the characteristics of interventions evaluated in RCTs supplemented by detailed analysis of the components of specialist services provided routinely in the United Kingdom through the National Health Service
Behavioural support (face‐to‐face, group)	Group discussion based on Maudsley model; typically weekly sessions, 90 minutes for first session then approximately 60 minutes on average after that; usually starting 2 weeks before the quit date and continuing for at least 4 weeks afterwards; led by one or two health professionals trained to NCSCT standard or equivalent; provided in a clinic setting to groups of between six and 30 smokers; premises, equipment and infrastructure support and supervision for practitioner, including ongoing monitoring of outcomes as per NHS Service and Monitoring Guidance or equivalent; free or low cost to user smokers making a quit attempt	The specification is derived from analysis of the characteristics of interventions evaluated in RCTs supplemented by detailed analysis of the components of specialist services provided routinely in the UK through the National Health Service
Behavioural support (telephone, pro‐active)	Provision of practical advice and emotional support and encouragement; 30–60 minutes for first session then approximately 15–30 minutes on average after that; usually starting before the quit date and continuing for at least 4 weeks afterwards; delivered by a health professional; free or low cost to the user; used by smokers making a quit attempt	The specification is derived from interventions evaluated in RCTs However, the specifics are less clear than for face‐to‐face support because there is less evidence from real‐world settings and some large studies have failed to show a benefit for this kind of support but it is not clear why 36
Behavioural support (text messaging)	Automated provision of practical advice and encouragement; multiple texts daily, tapering off after 1 month; usually starting up to 1 week prior to target quit date and continuing for at least 4 weeks afterwards; delivered by automated system; free or low cost to user; used by smokers making a quit attempt	The specification is based on evidence from RCTs. No evidence is available from real‐world evaluations
Behavioural support (printed materials)	Provision of practical advice and encouragement involving either one‐off book/booklets or multiple booklets; usually one‐off or delivered over a period of up to 12 weeks following the target quit date; provided by health professional or health promotion agency free of charge; provided in the absence of face‐to‐face support; used by smokers making a quit attempt	The specification is based on evidence from RCTs. No evidence is available from real‐world evaluations

NCSCT = National Centre for Smoking Cessation and Training (http://www.ncsct.org); NRT = nicotine replacement therapy; EQUIPT model = return on investment model for smoking cessation interventions in European countries; RCT = randomized controlled trial.

Table 2 shows interventions that were not included despite there being studies conducted on their effectiveness. It also provides justification for their exclusion. The main reason was an inability to make a confident assessment of the effect size. This does not mean that there was no effect: only that if there is an effect, its size remains uncertain.

**Table 2:** Smoking cessation interventions excluded from the analysis.

Intervention	Specification	Reason for exclusion
Bans on tobacco advertising 37	Bans on tobacco advertising vary in form and scope. In general. they prohibit marketing through particular channels such as television, film, posters or point‐of‐sale displays	Although it is possible that these would promote smoking cessation, it is not possible at this point to arrive at a reliable estimate of the effect size
Warnings on cigarette packets 38	Text and/or pictorial warnings on cigarette packets about the health effects of smoking	Although it is possible that these would promote smoking cessation, it is not possible at this point to arrive at a reliable estimate of the effect size
Standardized packaging 39	Requirement for cigarette and hand‐rolled tobacco packaging to conform to a fixed standard in terms of colours, fonts, size and shape, with no brand imagery	Although it is possible that this would promote smoking cessation, it is not possible at this point to arrive at a reliable estimate of the effect size
NRT (preloading) 40	Starting to use NRT for 2 or more weeks prior to the target quit date	Although there is some evidence that this improves success rates compared with starting NRT use on the quit date, the data are not yet sufficiently strong to warrant inclusion
Electronic cigarettes 41	Devices that use an electrical element to heat a liquid containing glycerol or propylene glycol, usually nicotine and often flavourings to produce a vapour that is inhaled. These vary widely in design and nicotine delivery	Although there is some evidence that these can help smokers to stop, the data are not yet sufficiently strong or consistent to permit a confident estimation of precise effect size. This is likely to change in the near future
Behavioural support (internet) 42	Websites and digital mobile applications designed to help smokers to stop	Although there are websites that have been found to aid smoking cessation, none of these are available and ones that are available have not been evaluated adequately. To date no firm evidence of the effectiveness of digital mobile applications is available

NRT = nicotine replacement therapy.

Table 3 shows the estimated reach and effect sizes of the interventions together with supporting reference and rationale. The effect‐size estimates cannot be compared directly with each other because they relate to different input metrics. However, they provide a broad indication of the probable impact of different contributions to a tobacco control strategy. In many cases they are subject to a wide degree of uncertainty, as indicated in Table 3. This is more the case for population‐level interventions, partly because the methods used to generate the effect sizes lead to greater uncertainty.

**Table 3:** Estimated reach and effect size of smoking cessation interventions.

(a) Interventions to promote quit attempts			
Intervention	Reach^a^	Effect size^b^	Rationale
Increases in taxation 43	100%	1.20	Based on consumption elasticity of −0.4 with assumption half of this being due to quitting and attributing all of this to an increase in incidence of quit attempts
Ban on smoking in public indoor areas 44	100%	1.10^c^	Based on study of effect of English smoking ban on quit attempt rates at the time it was implemented
Mass media campaigns 45, 46	100%	1.03^c^	Based on data on association between gross rating points and prevalence reduction in England, assuming that the reduction is achieved through an increase in quit attempts
Brief opportunistic advice by a physician 47	21%	1.40	Based on analysis of Cochrane Review specifically assessing effect on attempts to stop
Nicotine replacement therapy (NRT) for ‘reduce to quit’ 48, 49	12%	2.10^c^	Based on systematic review of RCTs of NRT for ‘reduce to quit’ supplemented by real‐world effectiveness estimate in England

(b) Interventions to improve success rates of quit attempts			
Intervention	Reach^d^	Effect size^e^	Rationale
Single form nicotine replacement therapy (NRT) [40]	5%	1.60	Based on Cochrane review of NRT for smoking cessation supplemented by real‐world effectiveness estimate in England
Dual form nicotine replacement therapy (NRT) 34, 40	2%	2.14	Synthetic estimate based on Cochrane review of single form NRT versus placebo multiplied by dual form NRT versus single form, supplemented by real‐world effectiveness estimate in England
Varenicline 34, 50, 51	5%	2.24	Based on Cochrane review supplemented by EAGLES trial and real‐world effectiveness estimates in England
Varenicline (extended duration) 50	1%	2.76	Synthetic estimate based on Cochrane review of varenicline versus placebo multiplied by effect in comparison of extended versus standard duration treatment
Bupropion 52	1%	1.60	Based on Cochrane Review of bupropion for smoking cessation
Nortriptyline 52	0%^f^	2.00	Based on Cochrane Review of nortriptyline for smoking cessation
Cytisine 50	0%^f^	3.98^g^	Based on Cochrane Review of cytisine for smoking cessation
Behavioural support (individual) 32, 53, 54	2%	1.40	Based on Cochrane review of individual behavioural support for smoking cessation, supplemented by real‐world evidence in England
Behavioural support (group) 34, 55	1%	1.88^g^	Based on Cochrane review of group‐based behavioural support for smoking cessation, supplemented by real‐world evidence in England
Telephone support (proactive) 56	0.5%	1.37^g^	Based on Cochrane review of pro‐active support for smoking cessation
Text messaging 57, 58	0.5%	1.63^g^	Based on most recent meta‐analysis of text messaging support for smoking cessation
Printed materials 59	1%	1.19^g^	Based on Cochrane review of printed self‐help materials for smoking cessation

a Reach refers to the proportion of smokers in England that are currently estimated to be exposed to the intervention.

b Effect size refers to the ratio of the prevalence of smokers who would be expected to make a quit attempt in a given year if the intervention were implemented compared with the rate if it were not implemented, other things being equal. Effect sizes are point estimates and subject to both a margin of error because of sampling variation in the studies, and also true variation as a function of variation in the delivery of the intervention.

c Estimate should be viewed with caution because of probably wide margin of error or variation due to implementation.

d Reach refers to the proportion of smokers in England who currently make a quit attempt in a given year who are exposed to the intervention.

e Effect size refers to the ratio of the proportion of smokers exposed to the intervention who are estimated to achieve 12 months of smoking abstinence compared with not receiving the intervention, other things being equal. Effect sizes are point estimates and subject to both a margin of error because of sampling variation in the studies, and also true variation as a function of variation in the delivery of the intervention.

f Nortriptyline and cytisine are not used in England but are available in other countries.

g Estimate should be viewed with caution because of likely wide margin of error or variation due to implementation. See Table 4 for caveats. RCT = randomized controlled trial.

Table 4 shows caveats attaching to reach and effect‐size estimates in Table 3. It should be noted that the effect‐size and reach estimates reported here may differ from the default values in the EQUIPT model. The estimates in this paper are the most up‐to‐date available at the time of writing.

**Table 4:** Caveats attaching to reach and effect size estimates of smoking cessation interventions.

Intervention	Caveat
Increases in taxation	Most of the evidence on the impact of taxation uses cigarette consumption as an outcome rather than smoking cessation. The estimate is dependent upon the tax increase leading to an increase in the cost of smoking. Evidence from a number of jurisdictions indicates that the effect of tax increases are often mitigated by pricing strategies of tobacco companies or actions by smokers such as trading down to cheaper products. Evidence does not support the claim by tobacco companies that raising taxes increases the purchase of illicit tobacco; the latter appears to be driven more by lax enforcement or increased opportunities for tax evasion. It is possible that at least part of the impact of tax increases is on the success of quit attempts, but evidence is lacking on this
Ban on smoking in indoor public areas	Different countries have implemented indoor smoking bans differently and that appears to have been associated with differences in their impact on smoking. It appears that comprehensive bans that are accompanied by strong publicity campaigns to secure popular support are important in securing adherence and motivating quit attempts. Evidence suggests that these bans have a one‐off effect around the time they are introduced. It is possible that at least part of the impact of indoor smoking bans is on the success of quit attempts, but evidence is currently lacking on this
Mass media campaigns	Mass media campaigns can be expected to vary substantially in their effectiveness depending on the content, intensity, patterning of delivery. The estimate provided here is based on traditional TV campaigns but other models are possible, e.g. setting up annual quitting events such as Stoptober 60. It is possible that at least part of the impact of mass media campaigns is on the success of quit attempts 61. There is currently insufficient evidence to estimate effect sizes for social marketing campaigns using digital media
Brief physician advice	The effectiveness of different types of advice may differ according to context. England has an extensive network of free stop‐smoking behavioural support and stop‐smoking medicines are reimbursed. This means that physicians only need offer support and refer to a specialist or provide a prescription. If smokers have to pay for treatment, the offer may be less effective. It is not clear whether brief advice from other health professionals has the same effect as from physicians
NRT for ‘reduce to quit’	It is not clear whether it is enough just to advise smokers who are not ready to stop to use NRT to help them cut down, or whether they need to be supervised and set a clear smoking reduction target in anticipation of setting a quit date in a few weeks or months.
Single‐form NRT	The evidence is strong and consistent that this increases the chances of quitting when used as part of a structured support programme but population data in England have found no evidence for a benefit when smokers simply buy NRT from a shop
Dual‐form NRT	The effect‐size estimate is a synthetic estimate based on data from placebo‐controlled trials of single NRT forms with data from comparisons between single‐form NRT and dual‐form NRT
Varenicline (Champix)	The effectiveness of varenicline appears to be very similar across different contexts and in different populations. Concerns about serious neuropsychiatric and cardiovascular side effects have not been supported by evidence from RCTs or very large observational studies. However, there has been relatively little research assessing the impact of varenicline in the absence of a structured behavioural support programme.
Varenicline (extended duration)	This is mainly based on one study 35. It is possible that the extended dose improved outcomes specifically for smokers who did not manage to quit on their target quit date but managed to quit later during the initial treatment period 62
Bupropion (Zyban)	The effectiveness of bupropion appears to be robust in multiple contexts but has not been tested in the absence of structured behavioural support
Nortriptyline	The confidence intervals on nortriptyline are wide because there are relatively few studies. This is a very inexpensive medication but needs careful monitoring by a health professional because of risk of overdose
Cytisine	The confidence intervals on cytisine are wide because there are still relatively few studies. It is not clear whether it is more effective than other pharmacotherapies but one large open‐label study found it to be superior to NRT in the context of a telephone support programme
Behavioural support (face‐to‐face, individual)	The evidence base for behavioural support is strong, but it is very difficult to estimate effect sizes because typically the comparison condition in studies also involves active support, and the more intensive the intervention condition typically the more intensive the comparison condition. The content and delivery of the behavioural support programme makes a difference to outcomes so it is important that the programmes conform to accepted standards and that they are delivered by appropriately trained and supervised staff 63, 64, 65, 66. There is some suggestion that the added benefit of behavioural support when given with stop smoking medication may be less than when it is the only treatment being provided 53 but this is not clearly the case
Behavioural support (face‐to‐face, group)	Although the effect‐size estimate for group‐based support is higher than for individual support, and success rates for group‐based programmes in England tend to be higher than for individual programmes, studies directly comparing the two have not shown group support to be superior 55
Behavioural support (telephone, proactive)	The effect size varies widely across studies and this form of support can present logistical difficulties when it comes to providing stop‐smoking medicines
Behavioural support (text messaging)	Evidence from the largest of the RCTs suggests that the effect may only be present in people not using stop‐smoking medication 67
Behavioural support (printed materials)	Studies to date have found that the effect is greater for tailored materials and is limited to contexts in which no other form of support is being used 59

NRT = nicotine replacement therapy; RCT = randomized controlled trial.

While the clinical interventions to support cessation have relatively low reach, the effect‐size estimates are more robust and they appear to be subject to somewhat less variation than the population‐level interventions.

## Discussion

### Summary of key findings

A wide range of smoking cessation interventions were identified for which it was judged possible to derive effect‐size estimates in relation to quit attempts or success of quit attempts. In terms of interventions to increase the rate of quit attempts these were: tax increases and associated measures to increase the financial cost of smoking, introduction of comprehensive public indoor smoking bans, mass media campaigns, brief opportunistic advice from health professionals and use of licensed nicotine products for smoking reduction. In terms of interventions to increase the success rate of quit attempts the interventions identified were: NRT (single form), NRT (dual form), varenicline (standard duration), varenicline (extended duration), bupropion, nortiptyline, cytisine, behavioural support (individual face‐to‐face), behavioural support (group face‐to‐face), behavioural support (proactive telephone), behavioural support (text messaging) and behavioural support (printed materials). While the effect‐size estimates are subject to a number of caveats, there can be a high degree of confidence that each of these interventions, if delivered as specified in a way that is appropriate to the context, will make a significant contribution to increasing population smoking cessation rates.

### Relationship between findings and previous research

Most of the interventions identified in this review are similar to ones shown previously to be important in reducing smoking prevalence. However, there were some additions, including differentiating single‐ and dual‐form NRT and standard‐ and extended‐duration varenicline. We also included interventions that are not used widely in western countries, but have been shown to be effective, such as nortriptyline and cytisine. An additional novel feature of this review is the estimation of reach and effectiveness of interventions specifically on quit attempts and quit success. This was necessary for the EQUIPT model, but more generally provides useful information for tobacco control strategy development in different countries that wish to focus attention on these different parts of the quitting process.

### Implications of findings

Combining the quit attempt and quit success effectiveness estimates can be used to generate an estimate of effect of interventions on cessation, and these estimates can be combined with reach estimates to arrive at figures for intervention impact. The Supporting information file contains an Excel spreadsheet with these calculations for England. In modelling the impact of combinations of interventions (e.g. mass media campaigns plus provision of single‐form NRT), some additional assumptions need to be made. The most important of these is that the intervention effects are independent of each other, and so the ratios representing effect sizes combine multiplicatively. Another is that the effect and reach are independent of each other. For example, if a mass media campaign leads more smokers to try to quit, increasing the reach of interventions aimed at increasing quit success rates, this does not influence the effects of those interventions.

An example ROI model (http://roi.equipt.eu) and its country applications using the data presented here are described elsewhere 68, 69, 70, 71. The ROI tools help decision‐makers to simulate the health and economic benefits compared to implementation costs under various investment scenarios; our reach and effect‐size estimates, therefore, are also useful ingredients to potential FCTC investment cases. The EQUIPT study has evaluated transferability of our estimates from England to other jurisdictions 72.

### Study limitations

The most important limitation of this review is that its effect‐size and reach estimates relate to specific contexts and implementations when in practice there will be large variation in both of these. Therefore, judgements will always need to be made as to whether, in a context to which the ROI model is being applied, the estimates are too high or too low. It would therefore be prudent to undertake sensitivity analyses using higher or lower values of the estimates to assess the relative impact of different combinations of interventions under different assumptions. One such analysis conducted has suggested that our estimates could provide the much‐needed input data in countries where context‐specific reach and effectiveness data are either not available or cannot be collected due to resource constraints 72. Undertaking sensitivity analyses will also be important to address the issue of varying confidence in the effect‐size estimates themselves. Thus, cytisine has a very large effect‐size estimate relative to other medications, but with much fewer data.

The review is also limited by the fact that each intervention's effect is estimated separately, and we do not have good information on interactions between interventions. The ROI modelling assumes that combining behavioural support and medication results in an effect that is the multiple of the effect‐size ratios of the components. The Cochrane review of behavioural support interventions suggests that this may not be valid, but we do not know. Neither do we know whether adding a mass media campaign to a tax increase leads to either or both interventions having a greater effect, a smaller effect or neither.

### Future research

The effect‐size and reach estimates in this paper will need to be updated as new findings emerge. It is problematic that very few countries have the kind of evidence needed to generate estimates specific to their context. The Framework Convention on Tobacco Control mandates countries that are party to the treaty to undertake the kind of population monitoring that would generate this kind of evidence 73, so it is to be hoped that more countries will begin to comply with this commitment.

## Conclusions

Tax increases, indoor smoking bans, brief opportunistic physician advice and use of NRT for smoking reduction can all increase population quit attempt rates. Quit success rates can be increased by provision of NRT, varenicline, bupropion, nortriptyline, cytisine and behavioural support delivered through a variety of modalities. These interventions can all make a contribution to national and regional strategies for increasing population smoking cessation rates. More countries need to collect the kind of data needed to be able to assess context‐specific parameters relating to smoking cessation to inform ROI models or use the data presented in this paper after an assessment of transferability.

### Ethical approval

None required for this analysis. However, the EQUIPT study, for which the current analysis was conducted, received full ethical clearance from Brunel University Research Ethics Committee.

### Declaration of interests

None, except for R.W., who has undertaken paid consultancy and received research funding and hospitality from companies that develop and manufacture smoking cessation medications (Pfizer, GSK, J&J). He is an unpaid adviser to the UK's National Centre for Smoking Cessation and Training.
